# A study on the practical logic and promotion path of agile governance of distorted health information on the internet: A review

**DOI:** 10.1097/MD.0000000000041897

**Published:** 2025-03-28

**Authors:** Yang Xiaowen, Zhang Shenzhong, Ding Jingjing, Ji Hanzhen, Pan Huiling

**Affiliations:** a Nanjing Medical University Library, Nanjing, China; b Institution of Medical Education Research, Nanjing Medical University, Nanjing, China; c Hospital Library, Nantong Third People’s Hospital, Affiliated Nantong Hospital 3 of Nantong University, Nantong, China; d Hospital Library, Taizhou, The Affiliated Taizhou People’s Hospital of Nanjing Medical University, Taizhou, China.

**Keywords:** agile governance, distorted health information on the internet, educational guidance, regulations and stipulations, scientific and technological empowerment

## Abstract

This paper aims to introduce the use of the agile governance theory in improving the identification and processing speed of distorted health information on the internet to ensure the security of public health information and maintain the authenticity of the network environment. Use literature research and content analysis to summarize the governance challenges of distorted health information and the shortcomings of governance responses. The governance of distorted health information encounters difficulties such as the rapid release of information, frequent updates, and the diversity and anonymity of information publishers. The current countermeasures against distorted health information mainly include community participation, technological means of monitoring, cross-platform cooperation, and data-driven governance. However, existing countermeasures still suffer from the governance process relying on community participation, poor cross-platform collaboration, limited application of algorithms, and insufficient enforcement of information regulation. These shortcomings affect the effectiveness and timeliness of countermeasures against disinformation, and there is an urgent need to further optimize and improve governance mechanisms. To assess the advantages of agile governance theory in the governance of distorted health information, and propose that the agile governance model of distorted health information contains three dimensions, namely, data empowerment, process reengineering, and role reconstruction, and that the implementation path is based on regulations and stipulations, scientific and technological empowerment, and educational guidance, and the interaction of these three dimensions achieves the ability of agile governance of distorted health information with rapid perception, flexible response, and continuous coordination.

## 1. Introduction

The widespread adoption of the Internet and information technology has made online access to health information an integral part of the public’s daily lives.^[1]^ However, the current Internet information ecological environment is exceptionally complex, and there exists a large amount of distorted health information. Distorted health information refers to information that is inconsistent with available scientific evidence, which poses a great threat to the physical and mental health of the public.^[2]^ Considering the rampant dissemination of distorted health information, the implementation of effective governance mechanisms and strategies has assumed paramount importance.

Current studies on the governance of distorted health information mainly encompass consumer rights and interests protection theory,^[3]^ social responsibility theory,^[4]^ information economics theory,^[5]^ news communication theory^[6]^ and social psychology theory.^[7]^ Some studies on governance reveal that: it is necessary to strengthen the regulation of false propaganda and protect the rights and interests of consumers; the companies are required to comply with ethics and provide truthful health information; it is imperative to improve the supervision of the quality and value of health information; the media regulation and sense of responsibility are enhanced; and the consumers’ psychology is studied to increase their health awareness and reduce their trust in distorted information.^[8–11]^ The studies on the governance of distorted health information show an increasing trend, and a series of governance strategies and methods have been put forward through in-depth study of the generation mechanism, dissemination pathway, and impact of distorted health information on public health, providing a good research foundation for this study. However, the current governance of distorted health information is facing a rapidly changing environment and a high degree of complexity and uncertainty, and the previous research measures have failed to better address the above problems.

Agile governance theory was first applied to software development, but its flexibility in responding to change and rapid iteration has led to its gradual expansion into multiple domains. For example, in data governance, agile governance improves data quality and management efficiency through flexible adjustment of data strategies and continuous feedback loops.^[12,13]^ In the field of risk management, agile governance helps organizations cope with uncertainty more effectively through cross-departmental collaboration and rapid response mechanisms.^[14,15]^ These applications provide the theoretical foundation for this paper’s exploration of cyber distortion health information governance, particularly the ability of agile governance to provide rapid response and flexible adaptation in the face of complex and changing environments.^[16,17]^

Therefore, this study introduces the agile governance theory based on the previous studies and focuses on the following 3 research questions: From the perspective of the governance challenges of distorted health information, what are the limitations and shortcomings faced by existing governance mechanisms in addressing public health and cybersecurity challenges? Based on the needs of distorted health information governance, we analyze the value and operability of agile governance in network distorted information governance from the aspects of key elements and core mechanisms. Explore the paths and frameworks for building an efficient agile governance model to adapt to the rapid dissemination and complexity of distorted health information.

## 2. Study process

In this study, the method of literature research was adopted to address the first research question, analyzing and exploring governance challenges of distorted health information. On the basis of the first research question, the practice logic and governance model for agile governance of the governance of distorted health information were explored.

The specific steps of literature research process are as follows: Identify the research question – governance challenges of distorted health information and formulate effective retrieval strategies; determine the inclusion criteria and select literatures relevant to the study; conduct a critical evaluation of the literatures to ascertain their validity; and summarize and analyze the target literatures by utilizing the content analysis and induction method.

### 2.1. Literature collection strategy

In this study, the foreign language databases such as Web of Science Core Collection, Scopus and ProQuest Dissertations & Theses, and Chinese language databases such as China Knowledge Network Database were searched for relevant literatures with a time range from January 1, 2013 to June 30, 2023. The literatures with relevant search terms such as (misinformation or disinformation or rumo*) and health appearing in title, abstract or keywords were retrieved. After de-duplication, 1627 foreign-language literatures and 275 Chinese-language literatures were obtained.

### 2.2. Literature inclusion criteria and quality assessment

**Figure 1.**
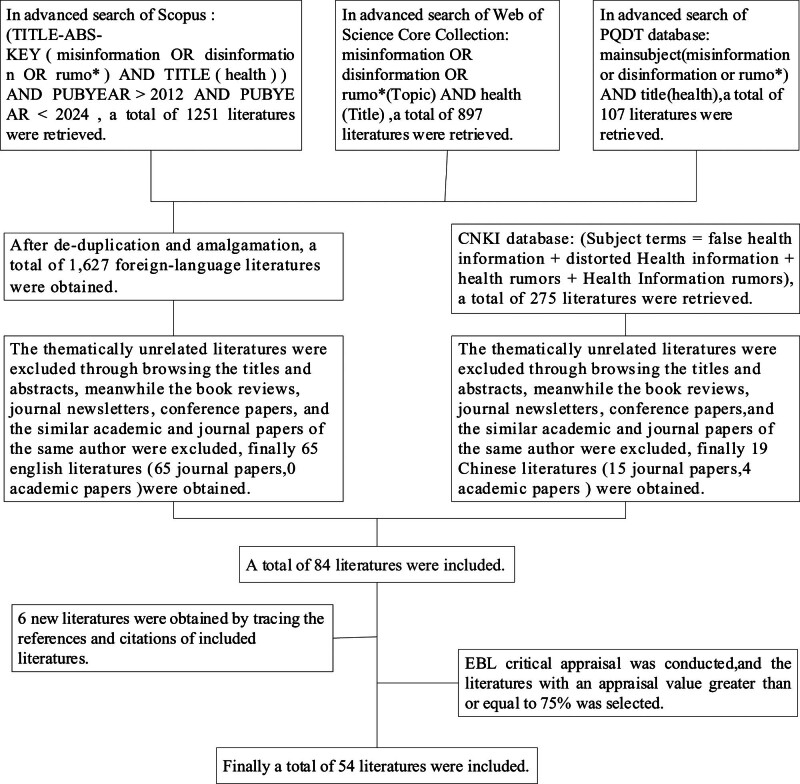
Search and screening process of literatures.

The screening criteria of literature were determined according to the reflections and suggestions on inclusion criteria in previous literatures,^[18,19]^ and the literatures were screened by browsing through the titles and abstracts of the literatures. The specific screening criteria were set as follows: the types of literatures were limited to Chinese and English journal papers, conference papers, and dissertations, including quantitative, qualitative and mixed-method studies, and the reviews and book reviews were excluded from this study; included literatures should involve governance challenges of distorted health information, and thematically unrelated literatures were excluded; and included literatures should meet the literature quality assessment criteria. Eighty-four literatures were obtained by screening, including 65 in foreign languages and 19 in Chinese. The references and citations of these 84 literatures were meticulously traced, resulting in the identification of 6 pertinent literatures. Finally, a total of 90 literatures undergo critical evaluation based on evidence-based librarianship, and those with evaluation values ≥ 75% were selected. Finally 54 literatures suitable for analysis were obtained, and the specific search and screening process is shown in Figure 1.

## 3. Results

For the 54 papers included in the analysis above, the governance challenges of distorted health information and the shortcomings of governance responses were derived through content analysis and generalization.

### 3.1. Governance challenges of distorted health information

With the development of information technology, the distorted health information is being released faster and faster, and the information is being updated and changed more frequently, which increases the difficulty in governance of distorted health information. With the advancement of science and technology, new diseases, new treatments, and new health products are constantly emerging, and the distorted health information is being constantly updated.^[20]^ The distorted health information also tends to change in various ways, involving the changes in content, form and platform of publication,^[21]^ which also poses a greater challenge to the governance of distorted health information.

Second, the publishers of distorted health information involve multiple forms such as individuals, organizations, business owners, media and social media, which makes it difficult for regulators to grasp the source of the information and take effective governance measures. Due to the anonymity of information publishers, they can evade accountability for publishing distorted information, which poses challenges in terms of tracing and removing such content. The anonymous publication may also attract a greater number of publishers to participate in, which will increase the difficulty of governance.^[22]^ Furthermore, the dissemination of distorted health information can be co-sponsored by multiple publishers originating from diverse regions, cultural backgrounds and interest groups, which will also increase the difficulty of governance.^[23]^

Third, the information authenticity and credibility judgments involve complex interactions among individual users, information sources, information content, information system components and information contexts, which makes credibility screening one of the most complex tasks in user information behavior. The distorted health information is strategically manipulated to confuse the truth, thereby increasing the authenticity of the information through the use of false evidence and propaganda, making it difficult for receivers to distinguish between the truth and the falsehood.^[24]^ The credibility of information is closely correlated with the reputation and qualifications of its publisher; however, the consumers are often compelled to invest substantial time and effort in understanding the reputation and qualification of the publishers.^[25]^ The receivers with varying backgrounds and information acquisition abilities show a difference in the evaluation and judgment of information.^[26]^

The above literature research and content analysis revealed that the difficulties encountered in the governance of distorted health information include fast information distribution, frequent updates, and diversity and anonymity of information publishers, this makes it difficult for a single governance mechanism to effectively deal with the problem of distorted health information. In order to better adapt to the changing characteristics of distorted health information and better coordinate the cooperation between different departments and different institutions, it is required to develop a set of pluralistic governance mechanisms with rapid response, dynamic adaptation, independent learning and good coordination.

### 3.2. Deficiencies in the governance response to distorted health information

Through the above literature research and content analysis, we found that the current countermeasures and deficiencies in the governance of distorted health information are shown in the Table 1.

**Table 1:** Governance responses and deficiencies of distorted health information

Governance responses	Shortcoming	References
Community engagement and trust-building are key to eliminating misleading information.	The governance process relies too much on community participation and fails to control the source of rumors in a timely manner.	^[27]^
Utilize media technology to track and censor information flow and improve public information literacy.	Failure to effectively stop the spread of information at its source.	^[28]^
Cross-platform cooperation and a sharing mechanism for algorithms to identify misinformation.	Cross-platform cooperation is currently not close enough to fully prevent the spread of conspiracy theories.	^[29]^
Utilize psychological methods to strengthen audience resistance to disinformation and enhance critical thinking.	Failure to adequately consider the support of technological tools for psychological defense.	^[30]^
Improvement of technological infrastructure and enhancement of information review mechanisms.	Frequent technical failures lead to governance failures.	^[31]^
Labeling and alerting emotional content to improve user recognition.	The filtering mechanism for emotional content is not perfect, failing to prevent the further spread of related information.	^[32]^
Enhance the provision of cultural sensitivity and mental health services.	Governance strategies lack effectiveness and relevance when confronted with religion-related information.	^[33]^
Enhance persuasive education for the public to improve the ability to recognize false information.	The influence of social media has not been fully utilized to counter disinformation.	^[34]^
Use artificial intelligence and machine learning technologies to recognize and manage disinformation.	AI technology, though effective, cannot fully cover all false information.	^[35]^
Enhance public health literacy education and push platforms to implement stricter content censorship.	The public’s ability to recognize false health information is limited.	^[36]^
Improve public trust in information and enhance psychological conditioning support.	Public anxiety is difficult to be quickly controlled.	^[37]^
Optimize information trustworthiness models to cope with the rapidly changing digital environment.	The means of assessing the credibility of information is still not refined enough.	^[38]^
Strengthen regulation of fake news through education and legal means.	The enforcement of existing legal mechanisms is limited.	^[39]^
Utilize platform algorithms to reduce the visibility of conspiracy theories while promoting authoritative information.	The application of algorithms has limited effectiveness and lacks human supervision.	^[40]^
Strengthen collaboration between scientific reporting and the media to reduce misleading stories.	Accuracy of scientific content in the media is lacking, and exaggerated reporting is still widespread.	^[41]^
Promote cross-party collaboration to reduce the negative impact of political polarization on public health.	Political factors are difficult to dissipate in the short term, resulting in false information remaining valid among specific groups.	^[42]^
Improve the ability to recognize misinformation through direct communication between physicians and patients.	Patients have limited access to correct medical information.	^[43]^
Enhance multilingual information dissemination and public education to improve information transparency.	The diversity of information dissemination is insufficient and fails to meet the needs of different cultural backgrounds.	^[44]^
Use deep learning models to identify disseminators of disinformation.	High complexity of the model, making it difficult to apply widely.	^[45]^
Enhance group management and promote health education in social networks.	Difficult to implement effective regulation in private groups.	^[46]^
Improve public health literacy and hold platforms accountable.	Insufficient regulation of the platform and low public awareness of disinformation.	^[47]^
The taxonomy should be continuously updated to address emerging health information challenges.	The tool has limitations in dealing with well-intentioned disinformation.	^[20]^
Encourage more physicians to deliver authoritative health information through social media.	Doctors have limited time and effort to fully cover disinformation dissemination.	^[21]^
Use social interaction trajectories and content analysis to improve the efficiency of identifying early disinformation.	The computational complexity of the framework is high, making it difficult to apply widely.	^[48]^
Improve regulation of scholarly publishing to ensure authoritative and reliable information.	The rapid proliferation of predatory journals is difficult to control completely.	^[23]^
Collaboration between media and fact-checking organizations is critical to enhance public trust.	The degree of freedom of the media varies from country to country, leading to mixed results of governance measures.	^[24]^
Use sentence vectors and FDA warning letters to match tweet content to improve detection accuracy.	The model has limited adaptability to other types of disinformation.	^[25]^
Integrate health communication training into medical education to encourage physicians to actively participate in public communication.	It has limited coverage and has not yet developed a broad social impact.	^[26]^

These studies have demonstrated the mechanisms of disinformation dissemination on online and social media platforms and the corresponding governance responses. Key governance strategies include utilizing technological means to identify disinformation, enhancing public media and health literacy, and strengthening platform accountability and management. However, studies have also revealed governance shortcomings, such as insufficient management of complex emotional content, loose cross-platform cooperation, limitations in the application of algorithmic models, and uneven enforcement due to differences across countries and platforms.

## 4. Discussion

The above studies have indicated that the governance of distorted health information requires a more flexible and adaptable governance model. The proposed agile governance theory provides a new way of thinking about the problem of complexity and uncertainty in the governance of distorted health information.

Agile governance^[49]^ originated in the field of software development at the end of the last century and was formally proposed by Qumer in 2007, aiming to cope with a situation of rapid change and uncertainty through adaptive and iterative approaches. It is currently applied in the fields of project management, organizational management, risk management and data governance. The core principles are flexibility, rapid response, iterative development and improvement, streamlining and optimizing processes, and multi-party collaboration and communication. An emphasis on rapid iteration and feedback loop can enable a team to respond quickly to changes and improve workflows and outcomes through learning and adaptation. An emphasis on collaboration and self-organization can facilitate information sharing and close collaboration among teams to increase efficiency and creativity. An emphasis on data-driven approaches can achieve better decision-making and greater efficiency.^[50]^

The reasons why agile governance theory is applicable to the governance of distorted health information, compared to current governance responses and what is not known, include the following, as shown in Table 2.

**Table 2:** Reasons why agile governance theory is applicable to the governance of distorted health information

Current governance deficiencies	Benefits of Agile Governance	Practical effects of agile governance
**Slow reaction speed** The traditional governance model, with its many levels of decision-making and cumbersome processes, results in a slow response to the spread of disinformation and an inability to cope with the rapid proliferation of information.^[35]^	**Rapid response and iteration** Agile governance enables rapid decision-making through streamlined processes, real-time data analysis, and the ability to take action to control the spread of information at its earliest stages of dissemination.^[51]^	**Improving response efficiency** Agile governance helps organizations identify and respond to unexpected distorted health information in a timely manner, reducing its impact on public health and public order.
**Difficulty in coping with a complex and changing environment** Traditional governance models are rigid and difficult to adapt to the complex and changing dissemination mechanisms of distorted health information.^[31]^	**Flexible adaptation** Agile Governance can flexibly adjust strategies and iteratively update governance tools in response to changes in information.^[52]^	**Dynamic adaptation** Agile Governance ensures that the governance strategy can readily respond to changes in distorted information in complex environments, maintaining the effectiveness of the response.
**Insufficient cross-sectoral collaboration** Inadequate cross-sectoral collaboration and limited information-sharing in the traditional model affect the efficiency of governance.^[29]^	**Cross-disciplinary collaboration** Agile governance encourages close collaboration among multiple actors (e.g., government, health sector, media, etc.) to form a linkage mechanism and facilitate information sharing.^[52]^	**Synergy** Collaboration enables information sharing and resource integration in different areas and improves the overall efficiency of governance.
**Reliance on manual and empirical judgment** Traditional governance tends to rely on experience and manual judgment, making it difficult to handle large amounts of data.^[36]^	**Data-driven decision making** Agile governance monitors and analyzes information in real time through big data and artificial intelligence to provide data-enabled decision making^[12]^。	**Precise and efficient** Utilizing data for monitoring and forecasting to enhance the scientific nature of decision-making and the precision of governance, and taking preventive and control measures in advance.
**Lack of feedback and continuous improvement mechanisms** Traditional governance models are often single measures that are difficult to adjust in real time to changes in information.^[38]^	**Continuous feedback and optimization** Agile governance continuously adjusts the strategy through feedback mechanisms to ensure that it is always responsive to the latest situation.^[53]^	**Dynamic optimization** Through continuous adjustment and optimization, agile governance can effectively respond to changes in the spread of disinformation over time, improving the timeliness and flexibility of governance.

**Figure 2.**
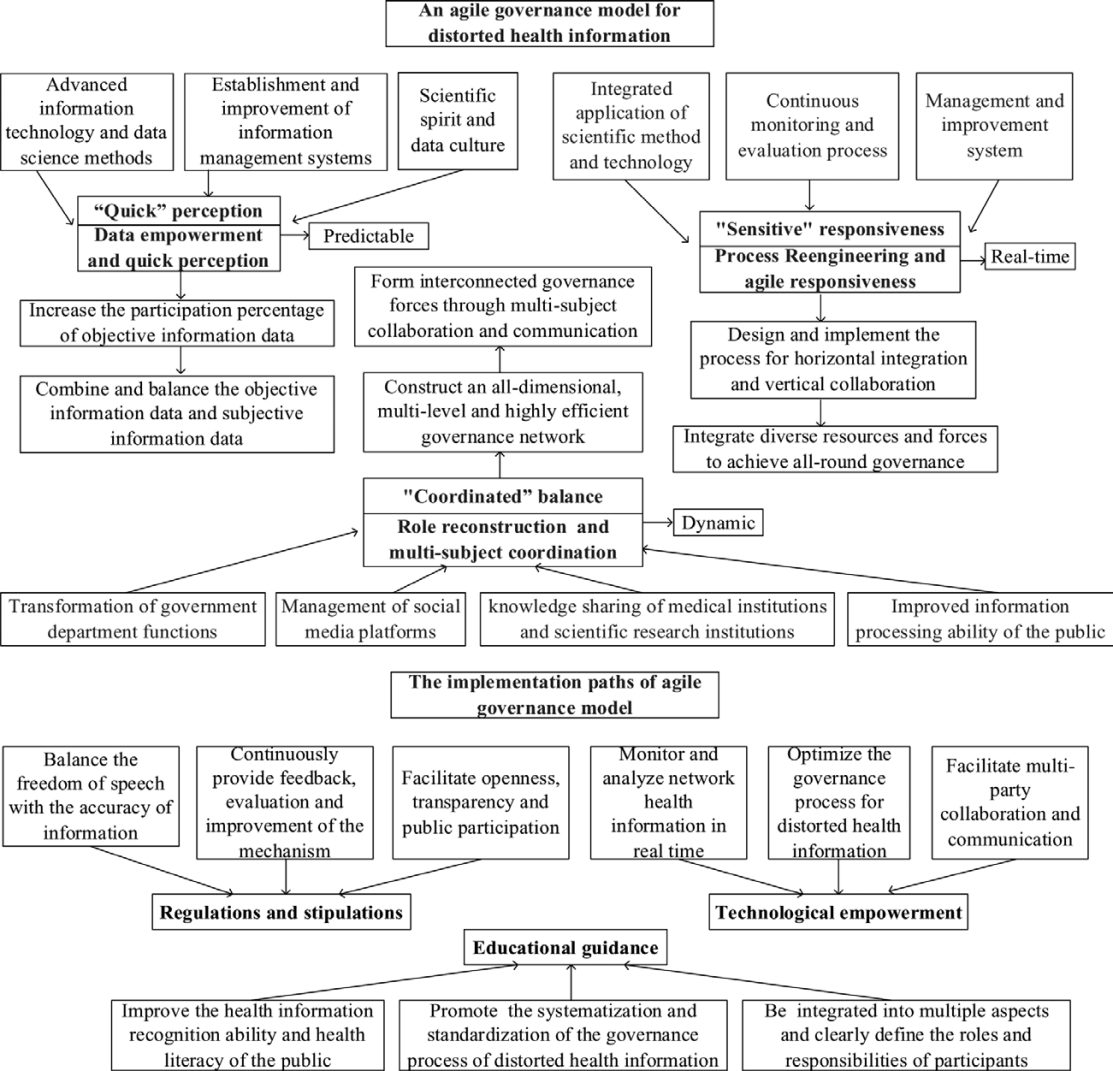
A framework for the agile governance of distorted health information on the Internet.

Therefore, we propose an agile governance model for distorted health information, which encompasses three aspects of the framework and three implementation paths, as shown in Figure 2.

### 4.1. Framework of agile governance model for distorted health information

#### 4.1.1. Data empowerment and rapid perception ability

The data include both objective and subjective information.^[54]^ The objective information is a type of information based on empirical data, scientific research, and specialized knowledge; the subjective information refers to personal opinions, experiences and emotions, etc. Enhancing the participation percentage of objective information can facilitate the rapid perception of distorted health information.

First, the objective information data based on empirical data and scientific research can intuitively and accurately reveal the generation, dissemination and impact of distorted health information, thus improving the speed and precision of our perception of distorted health information. Second, the empirical data can be continuously collected and analyzed to monitor the dynamic changes of distorted health information in real time, discover new ones promptly, and predict its possible propagation tendency to achieve a rapid response. Third, the objective information data of different types and sources, including user behavior data, social media data and expert assessment data, can reveal multiple aspects of distorted health information, such as content characteristics, dissemination paths, influencing factors, and effect assessment, thus providing a comprehensive and in-depth perception. However, elevating the participation percentage of objective information data does not mean neglecting the subjective information data. The subjective information data, such as user feedback, expert opinion, and social opinion, are also important resources for perceiving distorted health information. Compared with the objective information, the subjective information is more capable of revealing people’s emotional responses, value judgments, and behavioral motives, thus helping to understand the psychosocial mechanisms of distorted health information and improving the humanistic care and effectiveness of governance strategies. Therefore, it is necessary to increase the participation percentage of objective information data when respecting and utilizing subjective information data to achieve a more rapid, accurate and comprehensive perception.

#### 4.1.2. Process re-engineering and agile responsiveness

In the traditional governance model, the decision making need to go through multiple levels and links, which not only consumes a lot of time and resources, but also leads to delays and mistakes in decision-making. Streamlining and optimizing processes, carrying out flexible and iterative development and improving the governance process can make the governance strategies and tools more flexible and adaptable to quickly adapt and respond to rapid and unpredictable changes brought about by the generation, dissemination and impact of distorted health information

When designing and implementing the process re-engineering, it is necessary to fully understand and analyze the mechanisms of generation, dissemination and impact of distorted health information, determine the goals and strategies of governance, design suitable processes and structures, allocate appropriate resources and responsibilities, and establish effective coordination and monitoring mechanisms, etc.; it is necessary to comprehensively apply a variety of scientific methods and techniques, such as system analysis, process design, resource allocation, and risk management. When managing and improving process reengineering, it is necessary to continuously monitor and evaluate the operation and effect of the process, find and solve problems, and improve and optimize the process, etc.; it is necessary to establish and improve a set of scientific management and improvement system, such as quality management, continuous improvement, and learning organization. When evaluating and optimizing the process reengineering, it is necessary to regularly and systematically evaluate the effectiveness and impact of process reengineering, give feedback and learn from experience, optimize and upgrade processes, etc.; it is also necessary to establish and improve a set of scientific evaluation and optimization system, such as performance evaluation, experience learning, and process optimization.

#### 4.1.3. Role reconstruction and coordination of multiple subjects

The generation, dissemination and impact of distorted health information involve the participation of multiple subjects. Under the traditional governance model, the roles and responsibilities of the subjects are fixed and cannot adapt to the rapid changes in the network environment and social environment. Therefore, there is a need for role reconstruction, so that multiple subjects can flexibly adapt to new challenges and demands and fulfill their responsibilities more effectively. First, the government departments, as leading actors in governance, need to shift from the traditional command-and-control model to one that is more open, flexible and inclusive. New technological tools, such as big data and artificial intelligence, are utilized for monitoring and analysis of distorted health information. Other subjects are prompted to actively participate in the governance of distorted health information through policy guidance and regulatory supervision. Second, as the main means of information dissemination, the social media platforms need to be transformed from passive information providers to active information managers. it is necessary not only to audit and manage the information on the platform, but also to prevent and reduce the spread of distorted information through technical means, such as algorithmic recommendation and user behavior analysis. Third, as the main source of health information, medical institutions and scientific research institutions need to be transformed from closed knowledge producers to open knowledge sharers, they need to not only provide specialized health information and research results, but also communicate widely with the public through popularization of science and education to improve the public’s health information literacy. Finally, the public, as the receivers and disseminators of information, also need to undergo role reconstruction. They should change from passive information receivers to active information processors to improve their information literacy, identify and resist distorted health information, and participate in the reporting and governance of distorted health information.

It is also needed to carry out multiple subject coordination at the same time of role reconstruction. The aim is to construct an all-dimensional, multi-level and highly efficient governance network and form a coordinated governance force through the mutual collaboration and communication of various subjects. For example, the government departments have established information sharing and cooperation mechanisms with social media platforms, medical institutions and scientific research institutions to jointly monitor and deal with distorted health information. The social media platforms cooperate with medical institutions and scientific research institutions to provide and promote truthful and accurate health information. The medical institutions and scientific research institutions cooperate with government departments and educational departments to popularize and educate health information. The public participates in the reporting and governance of distorted health information through various channels such as social media, public forums, consumer associations.

### 4.2. Three pathways for the agile governance of distorted health information on the internet

#### 4.2.1. Regulations and stipulations: agility and efficient guarantee of the governance of distorted health information

Institutions play a central role in the governance of distorted health information, providing unified norms and principles for various stakeholders, ensuring consistency in the processing. Agile governance theory emphasizes the importance of cross-sectoral cooperation and collective wisdom, advocating for multi-party collaboration within the institutional framework to enhance governance effectiveness. This cooperation model is based on shared goals, principles, and support mechanisms, prompting different sectors to utilize their strengths to collectively address social challenges.

Agile governance focuses on finding a balance between freedom of speech and the accuracy of information. Institutions provide a clear framework designed to ensure that public freedom of speech is not excessively infringed upon, while also ensuring the accuracy of information. Additionally, agile governance theory believes that continuous improvement is key to successful governance, emphasizing optimization of the system through feedback mechanisms, continuous assessment, and improvement. This approach encourages open and transparent processing methods, providing a more effective path for future governance. Finally, agile governance theory values the openness and transparency of institutions. By improving the public’s understanding and trust in the governance of distorted health information through transparent governance mechanisms, compliance and acceptance are enhanced. Public participation and understanding contribute to the overall effectiveness of governance.

#### 4.2.2. Technological empowerment: the agile and diversified driving forces of distorted health information governance

Technology plays a crucial role in identifying and addressing distorted health information. With the help of big data and artificial intelligence, it’s possible to monitor online health information in real-time, swiftly identifying and recognizing distorted information. Data analysis can also predict the spread of distorted information, enabling early warning and prevention.

Technology provides flexible governance strategies that adapt to changes in the information environment. Using machine learning and deep learning technologies, information recognition models are continuously improved to address new forms of distorted information. Cloud computing and the Internet of Things assist in adjusting information monitoring and processing capabilities to meet the challenges of increasing information volume and faster dissemination.

Moreover, technology facilitates collaboration and communication among multiple parties, enhancing the fairness, transparency, and public participation in governance. Establishing public reporting systems and expert consultation platforms integrates expert knowledge to improve the accuracy of information processing. Collaboration with governments and businesses through API interfaces and data sharing helps combat distorted information collectively. The use of social media and online communities aids in promptly disseminating accurate health information, reducing misinformation and harm.

Technology also supports the iterative development and improvement of governance strategies and tools to meet the evolving needs of distorted health information governance. Data analysis and user feedback are used to evaluate and optimize governance methods. Blockchain technology aids in establishing mechanisms for tracing information sources and assigning responsibility, enhancing credibility. Virtual and augmented reality technologies are employed in health education and information dissemination, increasing public awareness and the ability to discern health information.

#### 4.2.3. Educational guidance: a key force to enhance the public resistance to distorted health information

Education is essential for enhancing the public’s ability to identify and respond to distorted health information. In the face of various forms of misinformation, education utilizes multiple methods such as health lectures, knowledge handbooks, discernment guides, and online courses to improve the public’s health knowledge and discernment abilities. Additionally, education emphasizes ethical constraints, encouraging the public to establish correct health concepts and lifestyles, thereby internalizing self-restraint and actively resisting distorted health information.

Education plays a key role in the systematic and standardized governance process of distorted health information. By establishing a long-term health information education and training mechanism, health education is integrated into various levels such as schools, businesses, and community services, making the governance process smoother and more efficient.

Education also promotes the integration of resources from different fields, such as medical health, psychological counseling, and law, providing the public with comprehensive and accurate health information. As a platform, educational training facilitates information exchange and experience sharing among the public, experts, government, and businesses.

Under the guidance of agile governance theory, education needs to flexibly adapt to environmental and demand changes, continually providing feedback and improvements, adopting a people-centered, transparent, and open governance approach. This method emphasizes respect and trust in recipients, encouraging active participation, while focusing on the actual effectiveness and impact of education. Agile governance advocates a bottom-up, people-centered approach, emphasizing listening to the public’s needs and voices, valuing the cultivation of critical thinking and autonomous learning abilities in the public, enabling them to make independent and wise judgments in the face of distorted information.

## 5. Conclusion

Based on the Agile Governance Theory, this study thoroughly analyzes the governance challenges of online distorted health information and the inadequacies of current governance countermeasures, and proposes an Agile Governance Model that can adapt to rapidly changing and complex environments.

The Agile Governance Model framework for distorted health information includes: Data empowerment and enhanced rapid perception capabilities, focusing on utilizing both objective and subjective data for effective information monitoring and analysis. Process re-engineering and strengthening agile response capabilities to quickly adapt to information changes by streamlining decision-making processes and enhancing flexibility. Role reshaping and coordination among various entities, including government, media, and social platforms, emphasizing adaptability and collaborative efforts.

The 3 primary paths for agile governance of online distorted health information are: Institutional regulation, ensuring consistent and efficient governance, balancing freedom of expression with information accuracy. Technological empowerment, leveraging advanced technologies like big data and AI for real-time health information monitoring and analysis, enhancing governance flexibility and accuracy. Education-led initiatives to bolster resistance against distorted information by improving public health knowledge and discernment skills, and promoting comprehensive health education.

Compared with existing studies, the research contribution of this paper is reflected in the following aspects:

First, most studies on the governance of online distorted information mainly focus on traditional governance models, laws and regulations, technical means and information literacy improvement. By introducing agile governance theory and highlighting the necessity of rapid response, flexible adaptation and cross-domain collaboration, it breaks through the limitations of existing research that relies on fixed governance frameworks, and provides a new theoretical perspective to deal with rapidly changing distorted information. Second, three major agile governance paths centered on institutional statute, technology empowerment and education leadership are proposed. These 3 paths provide a concrete implementation framework for rapid identification, agile response and multi-party collaborative governance of distorted health information, and make up for the shortcomings of existing research in the roles of multiple governance subjects and their interaction mechanisms. Third, it explores how to use technical means such as big data and artificial intelligence to realize real-time monitoring and early warning of distorted health information, which expands the discussion on technology application in current research and promotes the application and innovation of the data-driven governance model in the field of information governance.

Although this paper proposes a theoretical framework and implementation path for the agile governance model, the effectiveness and operability in practice are not yet supported by sufficient empirical research. Future research can further test the effectiveness of the agile governance model in practice through case studies and experimental validation.

## Author contributions

**Conceptualization:** Jingjing Ding, Hanzhen Ji, Huiling Pan.

**Funding acquisition:** Xiaowen Yang, Shenzhong Zhang.

**Methodology:** Jingjing Ding, Hanzhen Ji, Huiling Pan.

**Project administration:** Xiaowen Yang, Shenzhong Zhang.

**Supervision:** Jingjing Ding, Huiling Pan.

**Writing – original draft:** Xiaowen Yang, Shenzhong Zhang.

**Writing – review & editing:** Xiaowen Yang, Shenzhong Zhang, Jingjing Ding, Hanzhen Ji, Huiling Pan.
